# Paralytic Deformities of the Lower Extremities

**Published:** 1900-12

**Authors:** 


**Paralytic Deformities of the Lower Extremities.** By E. NOBLE SMITH. Pp. x., 99. London: Smith, Elder, & Co. 1900.

This is a book reviewing the general aspects of paralytic deformities of the lower extremities. It is chiefly an expression of the experience of the author in certain cited cases, rather than a systematic treatise on the subject, and in particular indicates deformities in medical affections which are amenable to surgical treatment. The author is of opinion (p. 65) that tenotomy of the tendon of a paralysed muscle may restore that muscle to function in certain cases.

